# Supplementary material for: Molecular Survey of Metastrongyloid Lungworms in Domestic Cats (Felis silvestris catus) from Romania: A Retrospective Study (2008–2011)
Source: Pathogens. 2020 Jan 26;9(2):80. doi: 10.3390/pathogens9020080 (PMC7168612; doi:10.3390/pathogens9020080)
Supplement: Supplementary file 1 [file pathogens-09-00080-s001.pdf]

Supplementary file 1

Lungworm species used in the phylogenetic tree construction

	**Host**	**Country**	**Accesion no.**
**Aelurostrongylus abstrusus**	x	Brazil	DQ029000
	x	Italy	EU034168
	x		DQ372965
	x		JX948745
	Felis catus	Italy	KM009116
		Romania	8
	Felis silvestris	Germany	KM506760
			KX518353
		Italy	KF751655
		Japan	KY774310
**Crenosoma vulpis**	*Vulpes vulpes*	Germany	KF836608
Dictyocaulus filaria	*Goat yeanling*	Turkey	KF471018
	Ovis aries	x	U37717
	Sheep	Iran	KC569369
	Soay sheep	UK	AY439020
Dictyocaulus viviparus	*Bison bonasus*	X	KM359415
		Poland	KF007341
	Bos taurus	x	U37718
	Cervus elaphus	x	KM359414
	Cattle	Sweden	AF105257
	x	Austria	KU891914
Metastrongylus elongatus	*Sus scrofa*	Estonia	AJ305378
			AJ305379
			AJ305380
			AJ305381
			AJ305404
		Uzbekistan	KF811486
Muellerius capillaris	*Capra hircus*	USA	AY679530
	Ovis aries	Canada	AY679528
	Ovis canadiensis	USA	AY679529
	Ovibos moschatus	Norway	KJ534592

Oslerus osleri	*Canis lupus*	Canada	JQ730005
Protostrongylus rufescens	Animals	USA	EU018485
	Caprinae	Uzbekistan	KF811499
Troglostrongylus brevior	Cat	Italy	KF241978
			JX290564
			KF751656
	Felis catus	Bosnia and Herzegovina	KT818789
		Italy	KM009117
		Romania	76
			223
			225
			304
	Felis silvestris	Romania	MF997544
	Lynx lynx	Central Europe	KY826439
		Japan	KY774311
